# Gonococcal Tenosynovitis With Abscess Formation: A Rare Presentation of Disseminated Infection

**DOI:** 10.7759/cureus.96815

**Published:** 2025-11-14

**Authors:** Afonso Granja, Cristina Ramos, Raquel Neto, Ana Nogueira, João Teixeira

**Affiliations:** 1 Family Health Unit (Unidade de Saúde Familiar - USF) Valbom, ULS (Unidade Local de Saúde) de Santo António, Gondomar, PRT

**Keywords:** disseminated gonococcal infection, empirical antibiotic therapy, gonococcal tenosynovitis, musculo-skeletal infection, neisseria gonorrhoeae, sexually transmitted infection (sti), surgical drainage, tendon sheath infection, wrist abscess

## Abstract

Gonococcal tenosynovitis is a rare but serious manifestation of disseminated *Neisseria gonorrhoeae* (*N.* *gonorrhoeae)* infection. It presents with localized inflammatory symptoms, including joint and tendon sheath involvement, and requires early recognition to prevent complications, including abscess formation, tendon rupture, and long-term functional impairment.

We report the case of a 40-year-old male who developed progressive swelling, erythema, and pain in his left wrist over 20 days, without significant response to oral anti-inflammatory treatment. Laboratory tests showed marked leukocytosis and elevated C-reactive protein (CRP), and imaging confirmed tenosynovitis with abscess formation involving the extensor compartment. The patient underwent open surgical drainage and was started on empirical piperacillin/tazobactam and vancomycin, which were later de-escalated to ceftriaxone following confirmation of *N. gonorrhoeae *via Gram stain and culture. He reported recent unprotected sexual intercourse, one week prior to symptom onset. Postoperatively, the patient improved significantly, with complete resolution of infection and full recovery of wrist mobility. He was discharged with outpatient orthopedic follow-up. Disseminated gonococcal infection (DGI) is an uncommon complication of gonorrhea that can manifest with arthritis, tenosynovitis, or dermatitis. The presence of abscesses may indicate a more severe or aggressive disease course. Diagnosis requires clinical suspicion, supported by imaging and microbiological confirmation. Prompt surgical intervention, combined with targeted antibiotic therapy, is critical to prevent complications such as tendon rupture and chronic disability.

This case highlights the importance of including gonococcal infection in the differential diagnosis of acute tenosynovitis, especially in sexually active individuals. It also underscores the need for multidisciplinary management and the pivotal role of primary care providers in early identification and timely referral to prevent adverse outcomes.

## Introduction

*Neisseria gonorrhoeae (N. gonorrhoeae) *is a common sexually transmitted pathogen, but disseminated gonococcal infection (DGI) is relatively uncommon, affecting only about 0.5-3% of patients with gonorrhea [1]. In most cases, dissemination occurs without obvious genitourinary symptoms, with many patients lacking a prior history of urethritis or cervicitis [2]. Once systemic, the infection has a biphasic course: an early “gonococcemia” phase characterized by fever, chills, malaise, tenosynovitis, and rash, followed by a later phase of localized suppurative arthritis [3].

Classic DGI presentations include the arthritis-dermatitis syndrome characterized by migratory polyarthritis, tenosynovitis, and a pustular rash. In some patients, only tenosynovitis occurs without frank septic arthritis [1,3]. DGI can affect multiple joints, but often involves the hands, wrists, knees, or ankles. Tenosynovitis is a common DGI manifestation, classically involving multiple tendons and especially affecting the flexor sheath of the wrist. Involvement of the extensor sheath can occur, but it is a rare presentation, especially in isolation [4]. Because many DGI patients lack genitourinary complaints, diagnosis often requires a high index of suspicion.

Early recognition and treatment are crucial. The diagnosis of DGI relies on microbiologic testing of both mucosal and disseminated sites. If tenosynovitis or an abscess is present, imaging (ultrasound or MRI) can identify fluid collections and guide drainage. Prompt targeted antibiotic treatment, including a third-generation cephalosporin, can prevent progression to purulent arthritis and other complications [4]. If tenosynovitis or abscess is present, especially in cases of delayed diagnosis, urgent surgical drainage and debridement are indicated along with the antibiotic regimen. Education of clinicians and patients regarding the possibility of DGI and the need for partner treatment can reduce morbidity [1-4].

## Case presentation

**Figure 1:**
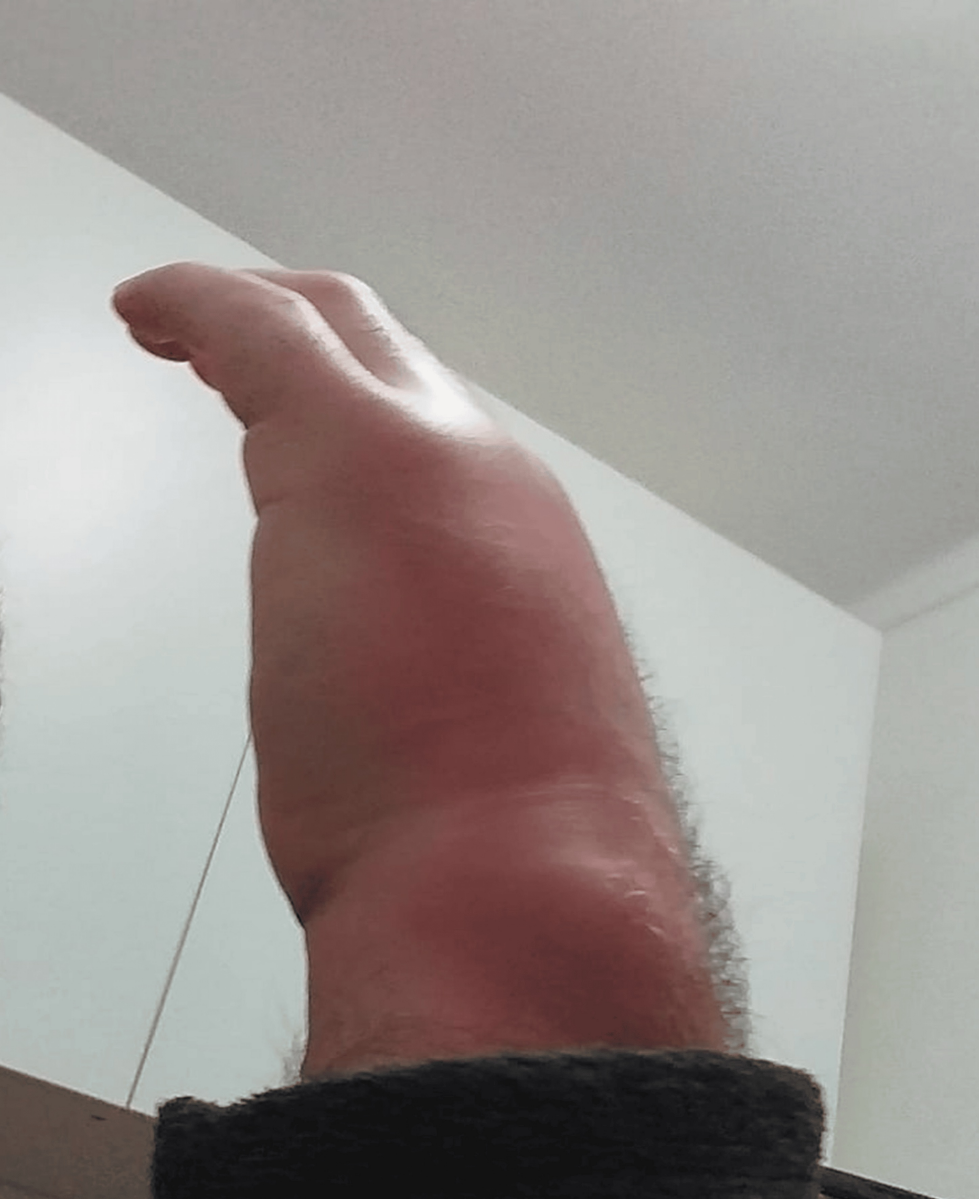
Initial presentation of the left wrist and hand Clinical photograph of the primary care center presentation showing marked swelling and tense erythema over the dorsal and ulnar aspects of the left wrist and hand, with limited extension of the fingers. These features corresponded to extensor‑compartment tenosynovitis and abscess, subsequently confirmed by ultrasound.

A 40-year-old, previously healthy male presented to an urgent care appointment at a primary care center with a 20-day history of progressive swelling, erythema, and pain in his left wrist and hand (Figure 1). He denied fever, chills, weight loss, or other systemic symptoms. On physical examination, the left wrist appeared visibly enlarged with overlying erythema and marked tenderness over its dorsal aspect, particularly around the extensor compartments. The passive and active range of motion in the wrist and hand was significantly reduced due to pain and swelling.

Over the course of these 20 days, the patient was evaluated on three separate occasions in both primary and urgent care settings. Initially, a gout flare was presumed and treated with oral nonsteroidal anti-inflammatory drugs (NSAIDs). Due to a lack of response, the treatment was changed to colchicine, which was later discontinued due to gastrointestinal intolerance (diarrhea). At the second evaluation, the therapeutic plan was adjusted to include oral NSAIDs and corticosteroids, which provided only transient symptomatic relief. Persistent symptoms and failure to improve prompted referral to the emergency department for further evaluation.

In the emergency department, laboratory workup showed elevated inflammatory markers, including leukocytosis and elevated CRP levels (Table 1).

**Table 1:** Relevant laboratory results on presentation to the emergency department

Laboratory parameter	Result	Normal Range
Leukocyte count	15.4 ×10⁹/L	4.0-11.0 ×10⁹/L
C-reactive Protein	159.29 mg/L	<5 mg/L

**Figure 2:**
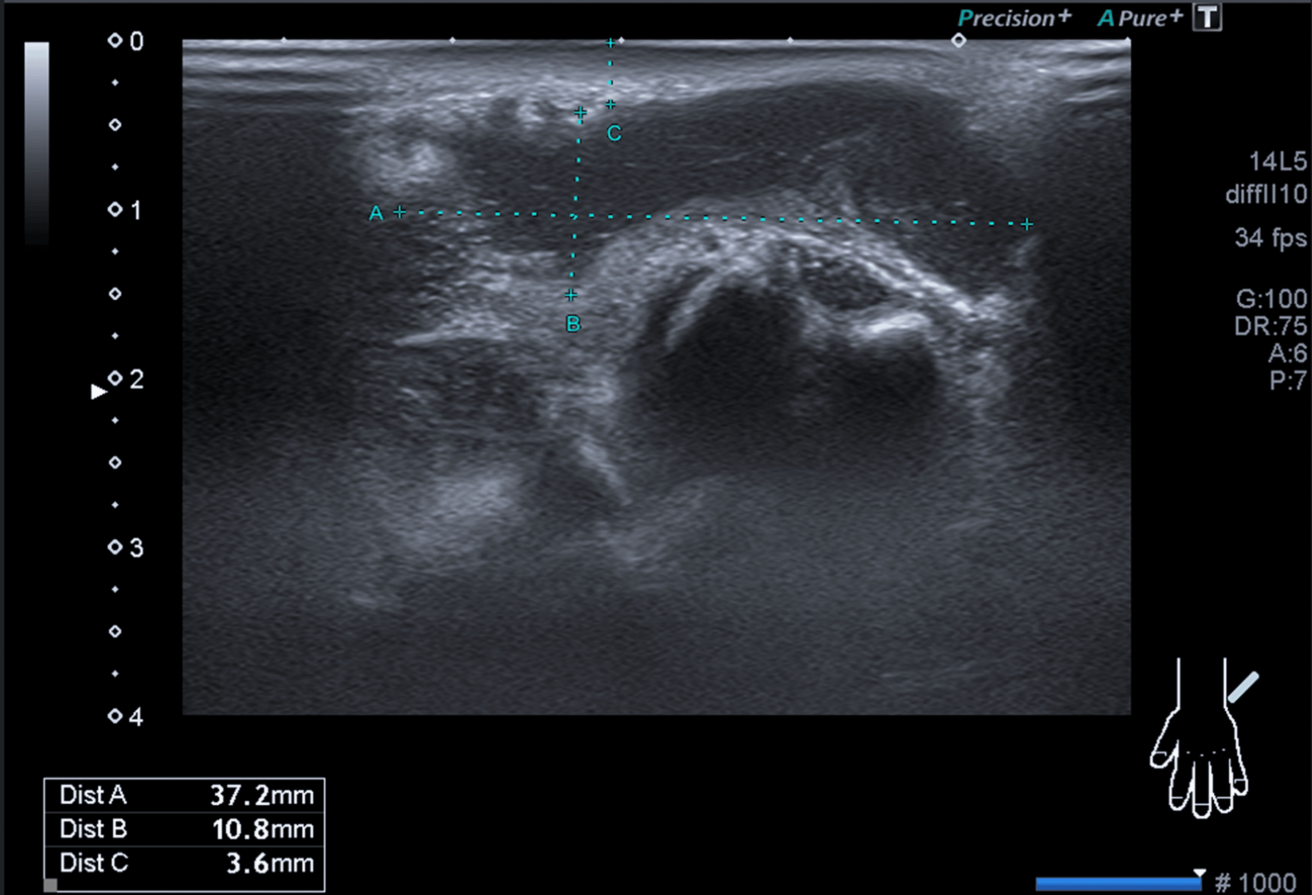
Extensor‑compartment abscess on longitudinal ultrasound Longitudinal musculoskeletal ultrasound of the dorsal left wrist showing an anechoic collection with internal echoes and septations along the extensor tendon sheath. Measurements are displayed as Dist. A (37.2 mm transverse width), Dist. B (10.8 mm anteroposterior depth), and Dist. C (3.6 mm from the skin surface). These findings confirmed the diagnosis of tenosynovitis with abscess formation and guided the surgical approach for open drainage.

Plain radiographs were unremarkable. Musculoskeletal ultrasonography revealed extensive tenosynovitis of the left wrist extensor compartments, with an associated anechoic abscess collection measuring 37.2 mm transversely and 130 mm longitudinally, extending from the distal forearm to the region of the fourth and fifth metacarpophalangeal joints (Figure 2). No concurrent arthritis or skin lesions were documented.

**Figure 3:**
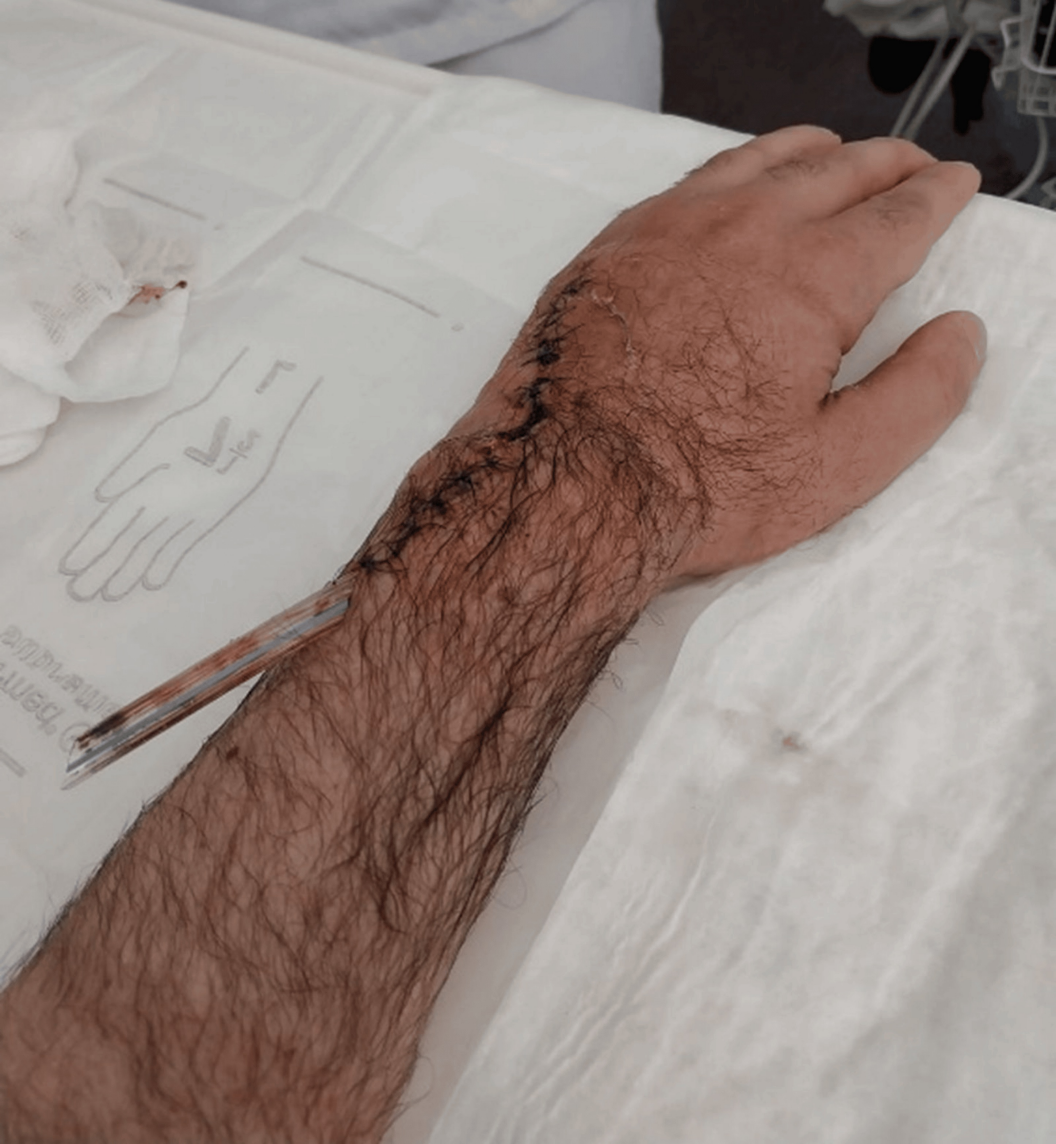
Post‑operative appearance after open drainage Photograph obtained after open surgical drainage, demonstrating the surgical incision along the dorsal wrist, the evacuated abscess cavity, and the in‑situ drain tube placed within the extensor compartment.

The patient underwent urgent open surgical drainage under general anesthesia (Figure 3). Blood cultures and intraoperative purulent material were collected and sent for Gram staining, microbiological cultures, and antimicrobial susceptibility testing, while abscess tissue was sent for histopathologic testing.

Empirical intravenous antibiotics were initiated with vancomycin and piperacillin-tazobactam. Within 48 hours, Gram staining revealed Gram-negative cocci, and intraoperative cultures grew *N. gonorrhoeae*, prompting a switch to intravenous ceftriaxone (1 g/day). Serologic screening for sexually transmitted infections (STIs), as well as urinary nucleic acid amplification testing (NAAT), returned negative for tested concurrent infections, including *Chlamydia trachomatis* (*C. trachomatis*). On further questioning, the patient disclosed a history of unprotected oral and vaginal intercourse approximately three weeks prior to symptom onset, but no prior genitourinary symptoms.

The patient showed rapid clinical improvement postoperatively, with declining inflammatory markers and resolution of pain and swelling. Repeat blood culture results were negative six days after surgery. He completed a seven-day course of intravenous ceftriaxone and was discharged with outpatient orthopedic follow-up. Public health authorities were notified for contact tracing, and partner identification, notification, and treatment procedures were initiated. He also received counseling on safer sexual practices during follow-up at his primary care center. As of July 2025, the patient had achieved full restoration of wrist and hand function, with no residual stiffness or complications, following a four-month course of physical therapy and rehabilitation.

## Discussion

Recent literature reports that disseminated gonorrhea can masquerade as isolated hand infections, with several recent reports underscoring the variable ways DGI can present with tenosynovitis or abscess. While less common, isolated extensor tenosynovitis has also been reported. Additionally, abscess formation is particularly unusual in DGI but has been documented and often signals a more severe clinical course. These findings often occur without obvious genital symptoms and may be accompanied by rash or arthritis. This variability of presentations means clinicians should keep DGI in mind for acute tenosynovitis, especially in younger patients or those with risk factors (multiple sexual partners, substance use, and HIV) [5-7].

Currently, gold standard treatment includes parenteral ceftriaxone 1g IV daily for 7-14 days, often supplemented with a 7-day course of oral doxycycline, to cover for possible *C. trachomatis* infection [4]. Whenever a tendon sheath abscess or frank purulence is present, prompt drainage and debridement are warranted. Repeated needle aspirations are preferred, while either arthroscopic or open surgical drainage should be reserved for complicated, severe, or refractory cases [8]. In all cases, specimens should be sent for Gram staining, cultures, and NAAT. If *N. gonorrhoeae *is confirmed, isolates should undergo susceptibility testing given the public health importance of resistance profiling [4].

With timely therapy, most patients with gonococcal tenosynovitis make a full recovery of function after drainage and antibiotics. In contrast, delayed or missed diagnoses can lead to serious morbidity with DGI frequently progressing from tenosynovitis to frank septic arthritis, with complications including tendon rupture, joint destruction, or need for surgical arthroplasty. Systemically, DGI can uncommonly cause osteomyelitis, endocarditis, meningitis, or septic shock [9].

This case highlights the crucial importance of obtaining a thorough sexual history incorporating the CDC's approach of the five P’s: Partners, Practices, Protection from STIs, Past history of STIs, and Pregnancy intention [10]. Clinicians should routinely ask about recent sexual contacts in patients with unexplained tenosynovitis, even if they deny dysuria or discharge. Emergency departments and primary care providers encountering unusual arthritis or tendon sheath infections should consider gonorrhea testing as part of their workup. Once a case is identified, public health measures are essential. Gonorrhea (including DGI) is a reportable infection in most developed countries; providers must notify local health departments so that contact tracing and treatment can proceed [4].

Although DGI is uncommon, its varied manifestations make it a “great imitator” in clinical practice. Family physicians and general internists, as frontline providers, play a key role in early detection. A young patient, especially if sexually active or with risk factors, presenting with fever and unexplained tenosynovitis or migratory arthralgias, should prompt inquiry about gonococcal infection. Early referral to orthopedics or infectious disease for suspected DGI can prevent the irreversible joint or tendon injury that can occur with delayed treatment.

## Conclusions

Gonococcal tenosynovitis with abscess formation is a rare but recognized complicated form of DGI. Recent literature emphasizes that any acute tenosynovitis in a sexually active patient should raise suspicion for gonorrhea. Timely imaging, microbiologic confirmation, and combined surgical-antibiotic management ensure optimal outcomes and prevent disability. This case report underlines the importance of considering DGI in the differential diagnosis of hand infections and of integrating sexual health assessment into routine musculoskeletal complaints.
